# Determinants of infarct progression and perfusion core growth in transferred LVO patients from remote regions

**DOI:** 10.3389/fneur.2024.1476796

**Published:** 2024-09-20

**Authors:** Michael Valente, Andrew Bivard, Bernard Yan, Stephen M. Davis, Bruce C. V. Campbell, Peter J. Mitchell, Henry Ma, Mark W. Parsons

**Affiliations:** ^1^Department of Medicine and Neurology, Melbourne Brain Centre at the Royal Melbourne Hospital, University of Melbourne, Parkville, VIC, Australia; ^2^Department of Neurology, Monash Health, Clayton, VIC, Australia; ^3^Department of Radiology, Royal Melbourne Hospital, University of Melbourne, Parkville, VIC, Australia; ^4^Department of Neurology, South Western Sydney Clinical School, Ingham Institute of Applied Medical Research, Liverpool Hospital, University of New South Wales, Liverpool, NSW, Australia

**Keywords:** ischemic stroke, CT perfusion, collateral flow index, collateral flow, infarct progression

## Abstract

**Introduction:**

Repeat imaging when regional and remote stroke patients arrive at a comprehensive stroke center (CSC) can delay endovascular thrombectomy (EVT). We examined which clinical and imaging parameters predict infarct progression and perfusion core growth during transport.

**Methods:**

We included patients recruited from 2017 to 2023 in a prospective database who were transferred from remote sites with large vessel occlusion, had CT perfusion imaging at the primary stroke center (PSC), and had repeat CT on arrival at the CSC demonstrating persistent occlusion. The key imaging characteristics were perfusion core change (rCBF < 30%) and ASPECTS change. Multiple and ordinal logistic regression analyses were used to assess the relationship between background clinical and imaging variables and the CT-perfusion core and ASPECTS on arrival. DEFUSE 3 criteria (ASPECTS ≥ 6, perfusion core < 70 mL) were used to define “favorable imaging.”

**Results:**

In 90 patients with CT perfusion at both PSC and CSC and persistent occlusion, the median time from onset to PSC presentation was 279 min (IQR 143–702). The median time from PSC presentation to CSC arrival was 243.5 min (IQR 186–335), and the median distance traveled was 186.5 km (IQR 101–258). Lower baseline ASPECTS (per point) was associated with a 7 mL increase (95%CI 2–11 mL) in perfusion core between scans (*p* = 0.004). The time from onset, the time between PSC and CSC, and the distance traveled were not significantly associated with either ASPECTS or perfusion core growth during transport. In total, 11 out of 78 patients (14%) had deterioration of initially favorable imaging profiles during transport.

**Conclusion:**

Perfusion core growth during transport was uncommon and most strongly associated with lower ASPECTS at the PSC. Initially, favorable PSC imaging May predict whether repeat imaging is necessary at the CSC.

## Introduction

Early reperfusion with endovascular therapy is of substantial benefit in ischemic stroke patients with large vessel occlusion (LVO). Due to geographical challenges, many patients May have delayed access. Systems must be optimized to improve how endovascular therapy is provided to patients who are from regional/rural areas without a local comprehensive (endovascular-capable) stroke center (CSC). Different models have been proposed for managing stroke patients, including direct bypass to the Comprehensive Stroke Center, the drip-and-ship approach (1), and pre-hospital stroke scoring scales for triage (2). For remote patients, the drip-and-ship model is the most appropriate. This approach involves initial imaging performed at the primary stroke center (PSC). Repeat imaging May be performed once patients arrive at the CSC due to concern for either infarct progression or recanalization. It is possible that unnecessary imaging May contribute to further recanalization delays (3).

Direct to endovascular suite transfer is generally ideal (3, 4). However, in practice, patients May be reassessed on arrival due to concerns regarding deterioration or improvement. This is primarily due to the significant cost and workforce required to activate the endovascular suite. Despite concerns regarding inter-transport deterioration, expansion of the ischemic core May be relatively uncommon (5) and unlikely to result in a patient becoming ineligible for endovascular therapy (6). In particular, a decline in the Alberta Stroke Program Early CT Score (ASPECTS) is uncommon in patients with high baseline ASPECTS and favorable collateral grades (5, 7, 8). Transport time also appears to have less association with infarct progression (5–7).

The framework of fast and slow infarct progressors has been understood for decades, but more recently has become a focus with the longer time windows for reperfusion therapies (9). Fast and slow progressors are terms that have been used to describe both baseline collateral status and infarct growth. Hypoperfusion intensity ratio (HIR) (Tmax > 10 s volume/Tmax > 6 s volume) and delay time index (DT > 6/DT > 2 s) quantify poor collateral flow and are associated with ischemic core growth (8, 10, 11). It is possible that a longer time to recanalization has less effect on outcomes in slow progressors (12).

We hypothesized that baseline clinical and imaging variables would be associated with whether remote patients continued to have favorable imaging profiles after long transfer (ASPECTS or perfusion core growth during transport).

## Methods

### Patients

**Figure 1:**
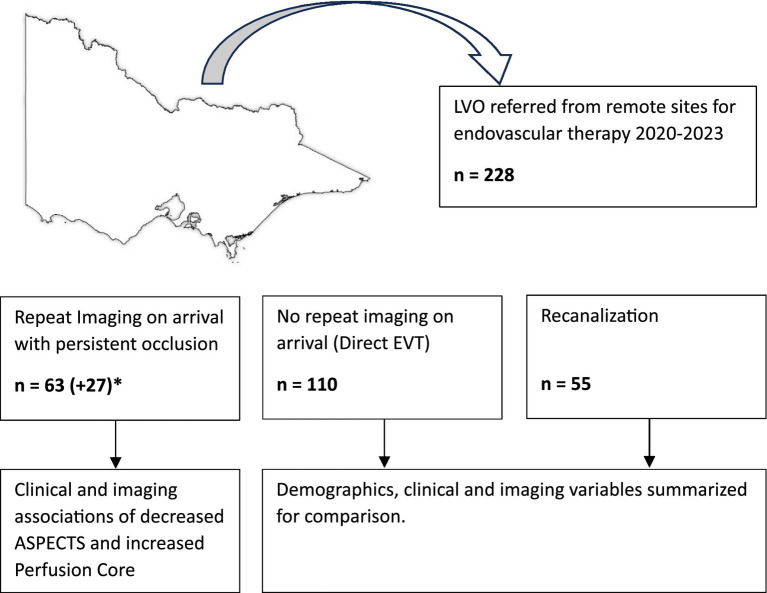
Study summary. *Retrospective cases from 2017 to 2020 identified.

Patients with LVO who were referred for endovascular therapy to the Royal Melbourne Hospital between June 2020 and February 2023 were prospectively recorded. Historical data from 2017 to 2020 was also reviewed for patients who had persistent occlusion on arrival. A summary of how patients were analyzed has been included in Figure 1. Data from 18 referral hospitals across Victoria and Tasmania were included. Multimodal stroke imaging was performed at the primary stroke center (CTB, CTA, and CTP) as part of a statewide telehealth protocol (13).

The clinical and imaging data of patients used for this study were extracted from The Monitoring of Stroke Endovascular Services study (MOSES) registry (HREC 2019.060). This registry includes patients being referred for endovascular thrombectomy (EVT) at the study center, a significant proportion of whom were transferred from regional centers. The baseline clinical data included age, sex, National Institutes of Health Stroke Scale (NIHSS), treatments received, premorbid modified Rankin Scale (mRS), and time from symptom onset. All data were collected in compliance with local ethics committee and institutional guidelines. Patients consented to the collection of their data. All management of patients (including whether they had repeat CT perfusion on arrival) was at the discretion of the treating physicians. Patients who had repeat CT perfusion at the CSC were considered to have “repeat imaging” for the purpose of this study. The data from this study are available from the corresponding author on reasonable request and with appropriate ethical approval.

CT perfusion was analyzed using the commercial software MIStar (Apollo Medical Imaging Technology, Melbourne, Australia). Automated maps were generated for delay time (a delay and dispersion corrected Tmax), cerebral blood flow (CBF), cerebral blood volume (CBV), and mean transit time (MTT). CTP penumbra was the total volume of delay time greater than 3 s (critical hypoperfusion volume) (14). The delay time index (DT index) was calculated on the PSC perfusion scan as delay time > 6 volume divided by delay time > 2 volume (11). In patients where raw perfusion data were unavailable for re-analysis using MIStar, historical documentation of thresholds was used to ensure that all patients were included. The ASPECTS evaluation was performed by the author (MV) with 5 years of experience as a stroke physician. DEFUSE 3 protocol (ASPECTS ≥ 6, perfusion core < 70 mL, mismatch volume > 15 mL, ratio > 1.8) was used to define “favorable imaging” (15).

### Statistics

The primary analysis focused on temporal changes in imaging and their association with baseline clinical characteristics in patients with persistent vessel occlusion. The key imaging characteristics were perfusion core change (CBF < 30%) and ASPECTS change in patients with repeat multimodal imaging (both remote PSC and repeat CSC imaging). Differences between patients who did and did not receive repeat imaging were summarized using medians and interquartile range (IQR). A paired *t*-test was used to compare the PSC mean perfusion core to the CSC mean perfusion core. In-table comparisons were performed using a two-tailed Fisher’s exact test or Mann–Whitney U test as appropriate.

For patients with persistent occlusion who had repeat CTP, multiple regression analysis was used to assess the relationship between background clinical and imaging variables and the presence of perfusion core on arrival. The dependent variable was set as the absolute change in perfusion core (CBF < 30%) and the independent variables included were PSC CT ASPECTS, PSC CTP core, PSC CTP penumbra, age, sex, onset to referral time, transport distance, transport time, NIHSS at onset, and thrombolysis usage. A further model was used to assess differences between occlusion types. Homoscedasticity and linearity were confirmed by plotting a scatter plot of the studentized residuals against the predicted values. Normality was assessed using a histogram with a superimposed normal curve and a P–P plot. The independence of residuals was assessed using the Durbin–Watson statistic.

Ordinal logistic regression was performed to explore associations with decreased ASPECTS on repeat scans. Change in ASPECTS was trichotomized into an ordinal dependent variable with three categories: decreased ASPECTS of 0–1, 2–3, and 4 or more. Independent variables included were PSC CT ASPECTS, PSC CTP core, PSC CTP penumbra, DT index, age, sex, onset to referral time, transport distance, transport time, NIHSS at onset, and thrombolysis usage. The independent variables were entered using a forward stepwise selection procedure. Bayesian information criterion (BIC) was used to assess the overall goodness-of-fit for each model. The assumption of proportional odds was assessed with full likelihood ratio testing comparing the fit of the proportional odds to a model with varying location parameters. Statistics were performed using SPSS.

## Results

Between June 2020 and Feb 2023, 228 remote patient transfers with LVO were referred for EVT. A total of 110 patients did not receive repeat imaging, 55 had repeat imaging with recanalization, and 63 patients had persistent occlusion on repeat CT perfusion (Figure 1). In total, 16 (19%) did not have CT perfusion at the PSC. The median age of transfers was 69 (IQR 58–79), with a NIHSS score of 14 (IQR 8–18), perfusion core of 17 mL (IQR 5–40), and penumbra of 96 mL (IQR 57–138). Clinical and imaging variables have been summarized in Tables 1, 2. Patients with recanalization during transport have been summarized in Supplementary Table S1. A total of 27 patients were identified from 2017 to 2020 with repeat imaging and persistent LVO for inclusion in the regression analysis (Supplementary Table S2).

**Table 1:** Clinical variables (transferred patients with persistent occlusion).

	Repeat imaging (*N* = 90)	Without repeat (*N* = 110)	*p*-value
Age (IQR), years	70 (60–79)	68 (57–78)	0.74
Male (%), *N*	43 (48)	60 (55)	0.39
NIHSS at onset (IQR)	14 (8–19)	14 (10–20)	0.42
Onset to referral (IQR), minutes	279 (143–702)	170 (114–367)	<0.05
Referral to repeat scan (IQR), minutes	243.5 (186–335)	n/a	n/a
Distance (IQR), km	186.5 (101–258)	161 (101–214)	0.08
Premorbid MRS 0–1 (%)	84 (93)	105 (95)	0.55
Thrombolysis (%), *N*	36 (40)	65 (59)	0.01
ECR (%), *N*	56 (62)	105 (95)	<0.01

**Table 2:** Imaging variables (transferred patients with persistent occlusion).

	Repeat imaging (*N* = 90)	Without repeat (*N* = 110)	*p*-value
**Occlusion site**			
M1 (%)	36 (40)	48 (44)	0.67
M2 (%)	14 (16)	9 (8)	0.12
ICA (%)	24 (27)	14 (13)	0.02
Tandem (%)	12 (13)	24 (22)	0.14
Basilar/PCA (%)	4 (4)	15 (13)	0.03
**Remote CT**			
ASPECTs (IQR)	8 (7–10)	9 (7–10)	0.4
Perfusion core, ml (IQR)	20 (3–44)	20 (8–39)	0.59
Penumbra, ml (IQR)	102 (61–159)	103 (70–138)	0.9
DT index	0.20 (0.12–0.32)	n/a	
**Repeat CT**			
ASPECTs (IQR)	8 (4–9)	n/a	n/a
Perfusion core, ml (IQR)	17 (4–52)		
Penumbra, ml (IQR)	111 (62–173)		

### Repeat CTP and persistent occlusion on arrival

Patients with persistent occlusion had a median initial PSC perfusion core of 20 mL (IQR 3–44 mL) and penumbra of 102 mL (IQR 61–159 mL) (Table 2). The mean increase in perfusion core from PSC to CSC imaging was 11 mL (95%CI −6 mL to 28 mL, *p* = 0.21). Of those with initially favorable imaging, 4 out of 78 patients (5.6%) had an increase in perfusion core above 70 mL during transport. In total, 7 out of 78 (9.0%) patients also had ASPECTS decrease during transport, resulting in a score less than 6 on repeat scans. When combined, 11 out of 78 (14%) patients had deterioration of initially favorable imaging profiles during transport.

### Factors associated with perfusion core growth on repeat CTP

**Figure 2:**
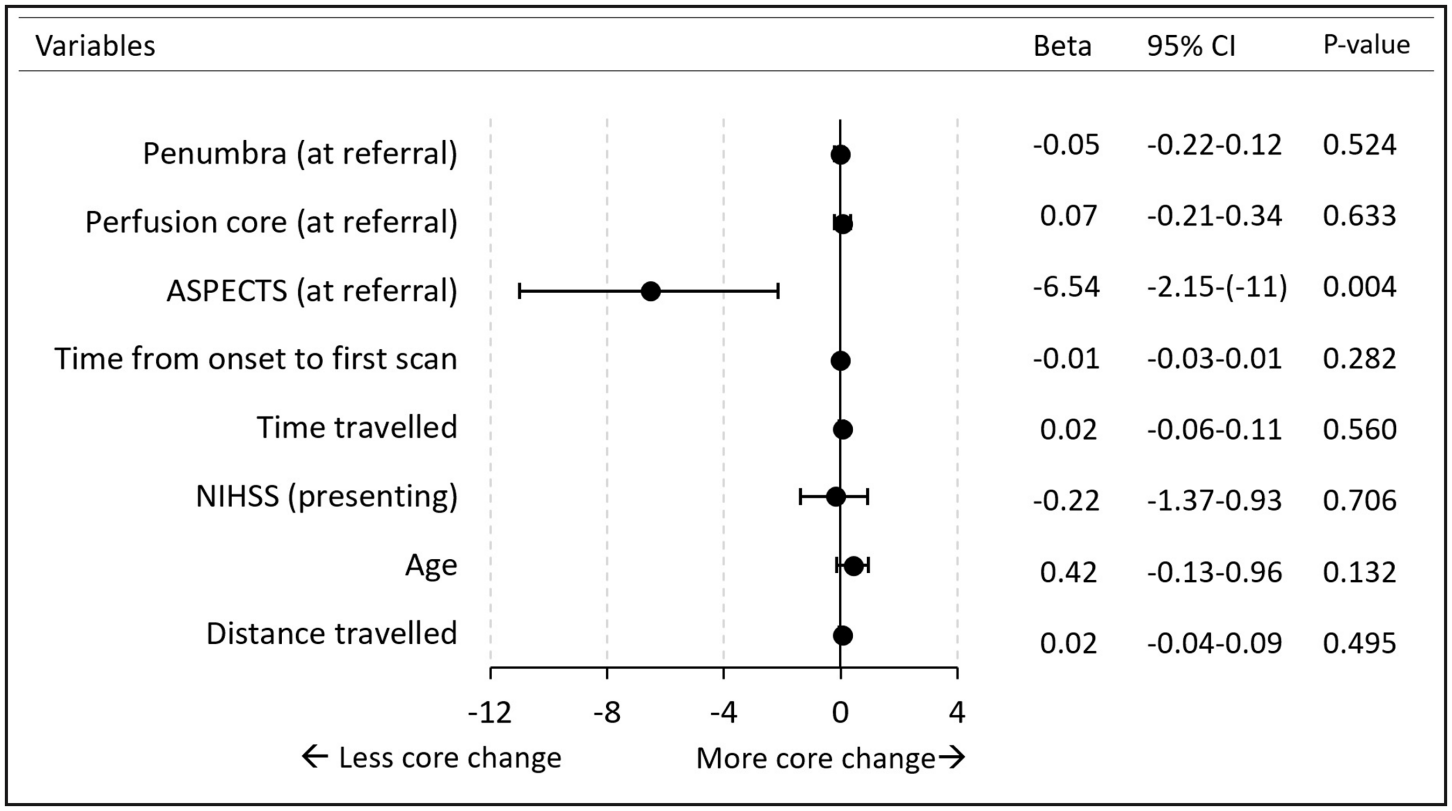
Forest plot. Multiple regression analysis of perfusion core increase. *Variables (HIR, lysis) excluded from the figure due to wide CI interval. No lysis beta 11 (95%CI −7 to 28). HIR beta −28 (95%CI −118 to 58). Time is measured in minutes. Penumbra/core measured in milliliter. Age is measured in years. Distance in kilometer.

Multiple regression analysis was performed to examine factors associated with an increase in perfusion core volume. The overall model (with all independent variables included) fitted with an R^2^ of 11%. ASPECTS was the only variable that significantly added to the association. A single point reduction in ASPECTS was associated with a 7 mL (95% CI 2–11 mL) increase in perfusion core on repeat scans (*p* = 0.004). Other independent variable relationships are summarized in Figure 2. There was no difference between occlusion types: MCA, ICA/Tandem, and posterior circulation (Supplementary Figure S1).

### Factors associated with ASPECTS progression on repeat scan

**Figure 3:**
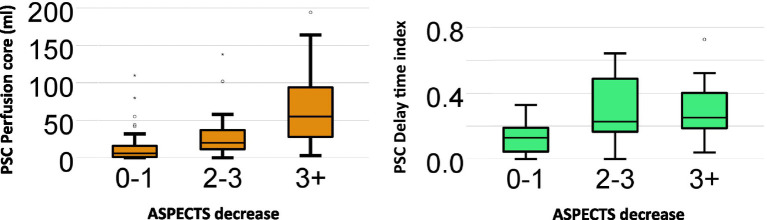
Box and Whisker plot: perfusion core and delay. Time index vs. ASPECTS decrease. Box and Whisker plot summarizes the relationship between initial primary stroke center (PSC) imaging (perfusion core and delay time index) and the absolute amount of ASPECTS decrease on the repeat scan at the comprehensive stroke center (CSC). The odds of decreasing ASPECTS with perfusion core (1 mL) was 1.02 (95% CI 1.01–1.04) and for DT index was 2.00 (95% CI 0.02–183).

The final ordinal regression model included the perfusion core and DT index. The perfusion core and DT index were significantly associated with ASPECTS progression, χ^2^(2) = 15.0, *p* = <0.001. The odds of decreasing ASPECTS with perfusion core (1 mL) was 1.02 (95% CI 1.01–1.04) and for DT index was 2.00 (95% CI 0.02–183). A summary of the perfusion core and DT index vs. ASPECTS increase is demonstrated in Figure 3.

## Discussion

The main finding of this study was a 14% rate of inter-transport deterioration of initially favorable imaging among patients with persistent large vessel occlusion. Although it was more likely for patients with longer transport delays to receive repeat imaging, time and distance traveled were not associated with either ASPECTS or perfusion core growth during transport. Early lower ASPECTS scores (<6) were associated with perfusion core growth in patients with persistent LVO (Supplementary Figure S2). ASPECTS deterioration was strongly associated with CT perfusion core volume at the PSC. The association between initial perfusion core volume and ASPECTS deterioration is best visualized in Figure 3, with the largest increases (>3 point ASPECTS increase) seen in patients with initial perfusion core above 50 mL. A higher delay time collateral index (indicating poorer collaterals) was associated with ASPECTS growth; however, the association was not as strong as the CT perfusion core.

Given the low rate of inter-transport infarct growth among patients with ‘favorable’ baseline imaging, the advantages of repeat assessment May not sufficiently outweigh the overall workflow delays. Patients transferred from remote regions provide an opportunity for endovascular laboratories to prepare their staff in advance of the patient’s arrival. When reassessment is planned, staff preparation May not occur until the decision to treat, which further compounds workflow delays. The MR CLEAN Registry (Multicenter Randomized Clinical Trial of Endovascular Treatment for Acute Ischemic Stroke in The Netherlands) demonstrated that each hour of delayed revascularization results in a 5% absolute reduction in the probability of a good functional outcome (16). Small institutional benefits that May be incurred by reducing angiography suite activation May be outweighed by the patient outcome benefits from minimizing revascularization delays. In addition to patient benefit, a direct-to-angio strategy May also be cost-effective (17).

Although repeat imaging should generally be minimized, low ASPECTS and high perfusion core (>50 mL) on remote scan predicted collateral failure and infarct progression. Interestingly, ASPECTS was a better predictor of collateral failure than other measures, which May be due to the negative effects of swelling on leptomeningeal collaterals (18). This is important due to recent large core trials (19, 20) demonstrating that endovascular therapy results in improved outcomes for patients with larger areas of established ischemic change. With these results being known, it is likely more remote patients will be transferred with lower ASPECTS and thus larger cores. Given our results, as well as others (5–8), patients with lower ASPECTS May be more suitable for repeat imaging as their collateral profile is more likely to degrade during transport.

CTP-based DT collateral index or hypoperfusion intensity ratio has been suggested as a way to triage patients who are remote to the ECR center to predict whether tissue will remain salvageable (21). In our patient group, the baseline DT index was associated with ASPECTS growth. However, the association did not appear to be as strong as the baseline perfusion core (CBF < 30%). The DT index was higher in those with ASPECTS growth but its association did not reach statistical significance within the multi-regression model. Nonetheless, the overall prediction model performed better with the addition of the DT index, which suggests that it can be used as a supplementary measure during initial patient triage [in addition to ASPECTs and perfusion core (CBF < 30%)].

Our findings are generally supported by other literature. Within an experimental model for collateral failure after 90 min, the initial degradation of collaterals plateaued (22). Within our study population, there was no association between perfusion core or ASPECTs growth and any time-related measure, suggesting that collateral failure is not a linear time-related process (predominantly occurring in the prehospital period). Minimal ischemic core expansion over time was also confirmed in a study of ultra-long transfers for EVT in Australia, with similarly low rates of inter-transport collateral deterioration among patients traveling 300 km+ (23).

Patients who received repeat imaging typically traveled further, had longer onset to presentation time, ICA occlusion, and less use of thrombolysis. They were, however, quite similar with regard to age, premorbid status, stroke severity, penumbra/core, and ASPECTS. Although an observational design could be prone to selection bias, observed similarities between groups are reassuring. However, this remains a limitation due to other unmeasured variables involved in physician selection, e.g., frailty. Another limitation is that all patients in the study were “selected” to be transferred for EVT, therefore potentially excluding patients with poor collateral circulation and/or large core at PSC for consideration of referral for EVT. Given recent large core trials this patient group May be a future area of interest. Larger, multicenter data would strengthen our findings further; however, PSC-level perfusion imaging was not widely performed outside our region.

## Conclusion

Repeat multimodal CT after long interhospital transfer demonstrated a relatively low rate of perfusion core growth among patients with LVO who had an initially favorable perfusion profile. Since changes in the CTP profile during transport are uncommon in the absence of recanalization, the benefit of repeat imaging upon arrival at the CSC is questionable. It May be more reasonable to consider repeat imaging only in patients with lower baseline ASPECTS (<6) or larger CT core (>50 mL). The need to repeat imaging should be weighed with workflow improvements in door-to-reperfusion times, which are strongly linked to better patient outcomes.

## Data Availability

The raw data supporting the conclusions of this article will be made available by the authors, without undue reservation.
