# Supplementary material for: Determinants of infarct progression and perfusion core growth in transferred LVO patients from remote regions
Source: Front Neurol. 2024 Sep 20;15:1476796. doi: 10.3389/fneur.2024.1476796 (PMC11449691; doi:10.3389/fneur.2024.1476796)
Supplement: Supplementary file 1 [file Data_Sheet_1.docx]

**Supplemental Figure One: Box and whisker plot – Occlusion type vs perfusion core absolute change during transport**


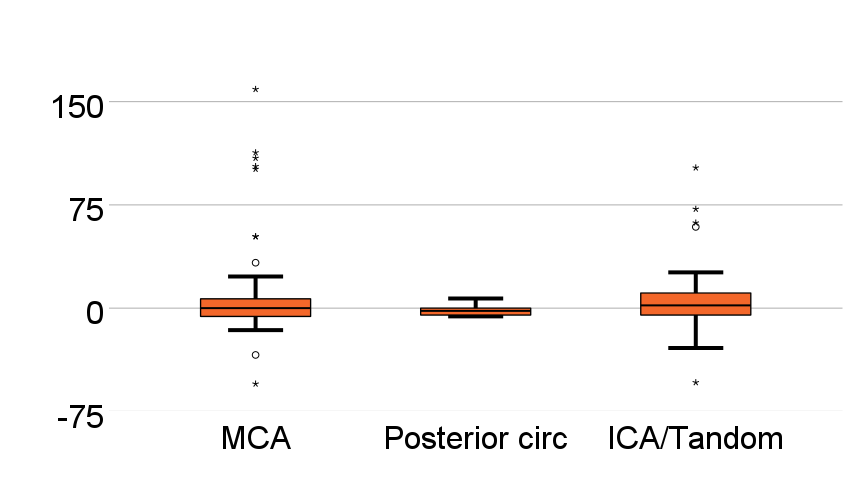


ICA/Tandem

**Perfusion core change (ml)**

Box plot demonstrating absolute change in perfusion core between PSC and CSC scan according to occlusion type. The occlusion type was not significantly associated with CBF change in the multiple regression model.

**Supplemental Figure Two: Box and whisker plot – Presenting ASPECTS vs perfusion core absolute change during transport**

**
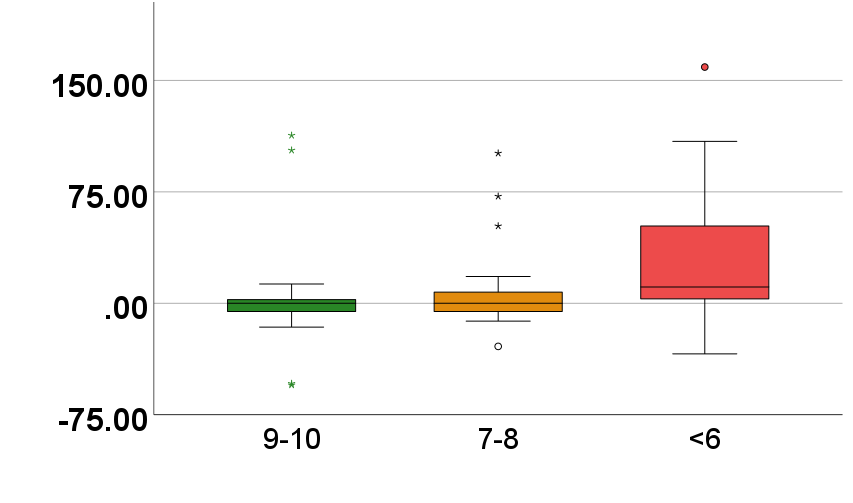
**

**Perfusion core change (ml)**

**Presentation ASPECTS**

Box plot demonstrating absolute change in perfusion core between PSC and CSC scan according to Presentation ASPECTS score (at the primary stroke center). A single point reduction in ASPECTS was associated with a 7ml (95% CI 2-11ml) increase in perfusion core on repeat scan (p=0.004).

**Supplemental Table One – Clinical characteristics of patients who recanalized**

|  | Recanalization  (N=55) | Without recanalization (N=173) | P- value |
| --- | --- | --- | --- |
| Age (IQR), years | 70 (62-79) | 69 (58-79) | 0.83 |
| NIHSS at onset (IQR) | 8 (5-14) | 14 (10-19) | <0.001 |
| Onset to referral (IQR), minutes | 194 (155-271) | 215 (137-514) | 0.03 |
| Premorbid MRS 0-1 (%) | 52 (95) | 162 (94) | 0.99 |
| Thrombolysis (%), N | 42 (76) | 88 (51) | <0.001 |
| Onset to needle (IQR), minutes | 192 (146-290) | 190 (134-274) | 0.56 |
| Occlusion site  -M1 (%)  -M2 (%)  -ICA (%)  -Tandem (%)  -Basilar/PCA (%) | 27 (49)  18 (33)  3 (5)  1 (2)  6 (11) | 74 (43)  22 (13)  30 (17)  29 (17)  18 (10) | 0.44  <0.01  <0.05  <0.01  0.99 |

**Supplemental Table Two - Retrospective cases compared to prospective cases**

|  | Retrospective cases  (N=27) | Prospective (N=63) | P- value |
| --- | --- | --- | --- |
| Age (IQR), years | 69 (55-79) | 70 (60-80) | 0.94 |
| Male (%), N | 10 (37) | 33 (52) | 0.25 |
| NIHSS at onset (IQR) | 11 (4-21) | 14 (9-19) | 0.55 |
| Onset to referral (IQR), minutes | 434 (89-826) | 264 (156-660) | 0.73 |
| Referral to repeat scan (IQR), minutes | 206 (145-273) | 265 (205-345) | 0.02 |
| Distance (IQR), km | 101 (67-188) | 226 (130-258) | < 0.01 |
| Premorbid MRS 0-1 (%) | 27 (100) | 57 (90) | 0.17 |
| Thrombolysis (%), N | 8 (29) | 28 (44) | 0.35 |
| ECR (%), N | 17 (63) | 39 (62) | 1.00 |
| Occlusion site  -M1 (%)  -M2 (%)  -ICA (%)  -Tandem (%)  -Basilar/PCA (%) | 10  1  7  7  2 | 26  13  17  5  2 | 0.11 |
| Remote CT  - ASPECTs (IQR)  - Perfusion core, ml (IQR)  - Penumbra, ml (IQR) | 8 (5-10)  20 (5-32)  113 (72-150) | 8 (7-10)  15 (3-53)  100 (59-168) | 0.51  0.86  0.55 |
| Repeat CT  - ASPECTs (IQR)  - Perfusion core, ml (IQR)  - Penumbra, ml (IQR) | 8 (5-10)  9 (4-38)  130 (72-169) | 7 (4-9)  22 (4-73)  106 (59-173) | 0.61  0.30  0.95 |
